# Experimentally-Verified Modeling of InGaAs Quantum Dots

**DOI:** 10.3390/nano12121967

**Published:** 2022-06-08

**Authors:** Alexander N. Kosarev, Vladimir V. Chaldyshev, Nikolay Cherkashin

**Affiliations:** 1 Ioffe Institute, 26 Politekhnicheskaya Str., 194021 Saint Petersburg, Russia; kosarev@mail.ioffe.ru; 2 CEMES-CNRS and Université de Toulouse, 29 Rue J. Marvig, 31055 Toulouse, France; nikolay.cherkashin@cemes.fr

**Keywords:** quantum dot, InGaAs, modeling, localized states, strain-stress field

## Abstract

We develop a model of an epitaxial self-organized InGaAs quantum dot buried in GaAs, which takes into account experimentally determined indium distribution inside the QD, its geometry and crystallography. The problem of solid mechanics was solved to determine the stress-strain field. Then, the parameters of the electron and hole ground states were evaluated by solving the problem of the quantum mechanics on the same mesh. The results of calculations appeared to be reasonably well consistent with experimentally recorded optical emission spectra for the QDs in the same sample. The experimentally-verified modeling reveals a bagel-like shape of the hole wave function at the ground state, which should considerably impact the optical and magnetic properties of the QDs. Such shape of the wave function is beyond the predictions of simplified models with uniform indium distribution.

## 1. Introduction

Semiconductor quantum dots (QDs) have attracted a lot of attention during the few past decades due to their ability to localize charge carriers in all three dimensions. In this sense, the QDs can be referred to as “artificial atoms” [1,2]. The artificially engineered localization of electrons and holes creates exciting possibilities for both fundamental and applied research. These include solid-state lighting, quantum information, energy harvesting, biological imaging, etc. [3,4].

The QDs can be produced in several different technological ways [5]. One of them is based on colloidal chemistry and provides free-standing QDs. Another one is based on epitaxial growth technology, such as molecular beam epitaxy (MBE) or metal-organic vapor phase epitaxy (MOVPE). Depending on the system, the epitaxial QDs are self-organized on the growth surface in the Stranski–Krastanow or Volmer–Weber growth modes. The self-organization process can be followed by an epitaxial overgrowth so that the QDs can be built in an active area of a semiconductor device in the bulk of the epitaxial film. In this paper, we consider such epitaxial self-organized QDs.

The self-organization technology is well developed for many material systems [6]. Among them, the InGaAs QDs are the most popular and practically important. In fact, the band gaps are equal to 0.4 eV and 1.5 eV in InAs and GaAs barrier, correspondingly. Therefore, the InGaAs QDs could potentially cover a very wide and important infrared optical range, including the common band of optical communications near 1.55 μm (0.8 eV). The typical InAs QDs self-organized on the GaAs (001) substrate during MBE and then overgrown by a GaAs barrier are the objects of interest in our study.

The engineering of the electron and hole localization in epitaxial self-organized QDs should take into account several phenomena, which apply strong restrictions on the energy and spatial structure of the localized states. First of all, the Stranski–Krastanow growth mode requires a lattice mismatch between the materials of the QD and the barrier. Then, the self-organized QD is mechanically stressed, especially if it is buried in the film bulk. The mechanical stress changes the localization potential and results in a strong blue shift of the optical emission from the QD when compared to the unstressed InGaAs [7,8].

Another blue shift originates from quantum confinement. This shift cannot be reduced below a certain amount if coherency of the QD interfaces is required. For the given lattice mismatch between the QD and barrier materials, the QD should generate misfit dislocations when its size exceeds a critical value [9,10]. The formation of the dislocation loops at the QD interface is normally unacceptable for any electronic or optoelectronic applications.

The self-organization of the QD and their overgrowth are provided by the migration of the atoms over the growth surface and intermixing of the surface atoms with the atoms in the underlying layers [11,12]. Therefore, certain In-Ga intermixing and QD reshaping are intrinsic processes associated with the formation of InGaAs QDs in the bulk of the epitaxial film. Both phenomena are important for the localizing potential and quantum states of electrons and holes [8].

The evaluation of the actual shape and indium distribution within a QD buried in the bulk of GaAs is an extremely hard task, which requires sophisticated characterization techniques. The authors of Ref. [13] reported the first direct elemental mapping of the strong lateral and vertical indium composition modulation of islanded uncapped In_x_Ga_1−x_As thin films grown on GaAs (001). The problem of the indium segregation and reshaping of the QDs during the overgrowth was addressed in Ref. [14] by using cross-sectional scanning-tunneling microscopy. The authors determined the actual geometry and revealed an indium gradient from the base to the top of the QDs. A three-dimensional image of indium distribution has been reconstructed in Ref. [15] by using a high-angle annular dark-field scanning transmission electron microscope. The authors also observed indium segregation near the QD top. The high-resolution TEM images and dark-field electron holograms were analyzed in Ref. [16], which results in a precise description of the shape and 3D indium distribution in non-truncated InGaAs QDs.

The precise structural investigations were not followed by appropriate quantum-mechanical calculations, which could be verified by optical study. Till the precise structural data were available, the calculation of the stress-strain field followed by solving the quantum-mechanical problem was performed on a model of a pyramid with (001) base and {110} facets [7]. The actual reshaping during the overgrowth was taken into account in Ref. [8]. However, Refs. [7,8] and other papers (see, for instance, [17,18,19]) utilize a simplified model with uniform distribution inside the InGaAs QD.

In this paper, we consider the experimentally deduced In distribution and the actual shape of InGaAs QDs, calculate corresponding potentials taking into account the stress-strain field, and solve the quantum-mechanical problem for electrons and holes. The results are verified by comparison with an experimental optical emission spectrum from the ensemble of QDs in the same sample. The structural and optical data appeared to be well consistent within the model. The model reveals an unusual spatial distribution of the wave function for the ground state of the localized holes.

## 2. Materials and Methods

The research in this paper has been done with reference to the buried non-truncated pyramidal InGaAs QDs, which were self-organized using Stranski–Krastanow growth mode by MBE at 460 °C The growth details can be found in Ref. [16].

The geometry and chemical composition of the InGaAs QDs was previously studied by geometric phase analysis (GPA) of high-resolution transmission electron microscopy images and dark-field electron holography (DFEH) applied in Lorentz mode [16]. Both methods provided 2D strain tensor maps with at best a 1 nm spatial resolution that may question the interpretation of the interface’s abruptness. Here, TEM investigations were carried out using I2TEM–Toulouse, a HF-3300 (Hitachi High-Tech Corporation, Tokyo, Japan) TEM operating at 300 kV, equipped with a cold-field emission source, an imaging aberration corrector (CEOS B-COR), a multiple biprism system, and a 4k CCD camera. Such a TEM allows for DFEH experiments with the sample holder position within the objective lens. As a result, image aberration correction can be applied and an ultimate hologram fringe spacing of 0.1 nm can be obtained which is essential for the analysis of extremely thin interfaces and layers [20,21]. The interference fringes of holograms are substantially less sensitive to focus, lamella thickness inhomogeneity, and chemical changes than HR-TEM images [22]. In this experiment, the HR holograms were recorded using diffraction vectors g = 111 and g = 004 over a QD sectioned by a 10–20 nm-thick cross-sectional (110) lamella. Several QDs were analyzed. Since the results are similar, we present the data for only one QD.

**Figure 1:**
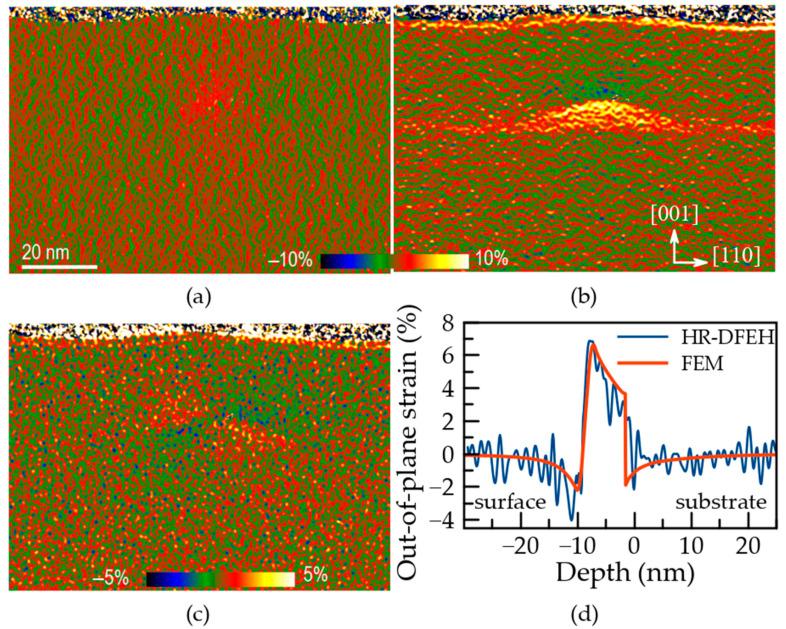
(**a**–**c**) Strain tensor components defined with reference to GaAs lattice and (**d**) vertical out-of-plane strain profile passing through the QD apex in (**b**) obtained by HR-DFEH: (**a**) in-plane, (**b**) out-of-plane, (**c**) shear strain.

Figure 1 shows the in-plane (a), out-of-plane (b) and shear strain (c) tensor components obtained by HR-DFEH with reference to GaAs lattice with a 0.6 nm spatial resolution and 0.4% precision. To begin, we observe that the results obtained thus far are qualitatively consistent with those previously obtained using alternative approaches [16]. Inside, below, and above the QD, tensile in-plane strain is visible, attaining higher values near the QD apex (Figure 1a). The QD’s pyramid-like shape is mirrored in the tensile out-of-plane strain distribution (Figure 1b). The Poisson reaction of in-plane stretched GaAs lattice is indicated by compressive out-of-plane strain measured immediately above the QD apex. The presence of the wetting layer is shown by a 2D layer around the QD with zero in-plane strain (Figure 1a) and a quickly decreasing tensile out-of-plane strain (Figure 1b). The shear strain distribution has an inverse mirror symmetry with respect to the central vertical axis of the QD (Figure 1c). When comparing the sharpness of the QD interfaces in Figure 1 to that obtained in [16], it is obvious that the interfaces are indeed abrupt, and that the diffused character found in [16] is primarily due to a lower spatial resolution of the methods utilized there for the strain measurement.

The experimentally-verified model of the InGaAs QD shape, atomic structure, and chemical composition was utilized in calculations by using the finite element method (FEM) in the following way. First, we solved the problem of linear solid mechanics for the reference QD embedded within a 10 nm-thick lamella. For this, we employed the same model input parameters as in [16] for a QD form, dimensions, base orientation, and 3D indium component distribution. The set of the material parameters for the In_c_Ga_1−c_As solid solution is listed in Table 1. The cell size was 100 × 100 × 110 nm^3^ with the InGaAs QD and wetting layer embedded in the GaAs continuum. We used the free-surface boundary condition on the cell top and zero displacements at the bottom. We denote *z* as the growth direction [001] and select the *x* and *y* in-plane axes along crystallographic directions [100] and [010]. The out-of-plane strain profiles obtained along the vertical line passing through the QD apex demonstrate a very good agreement between the experiment (Figure 1d, black line) and the model (Figure 1d, red line). Note that, unlike in [16], we have drawn a strain profile straight from the model, without altering it for the effect of the experimental spatial resolution. We show that a subnanometer spatial resolution has a small effect on the derived strain maps in this way.

The following geometry revealed from the TEM investigations is utilized for the FEM calculations of the reference InGaAs QD. The QD has a four-fold pyramidal volume of *H* = 9 nm height and a squared base with side lengths of *L* = 28 nm parallel to in-plane <010> directions. The pyramid has {203} facets with <332> edges. The experimentally determined indium distribution $C_{QD}^{In}(x,y,z)$ in the QD is analytically approximated as follows:

(1) $$C_{QD}^{In}(x,y,z)=C_{base}^{In}+\left[f_{facet}^{In}\left(z\right)-C_{base}^{In}\right]\frac{z}{L/\sqrt{2}-x-y}\frac{L}{\sqrt{2}H},$$

where the origin coordinate (0,0,0) situates at the center of the QD base and

(2) $$f_{facet}^{In}\left(z\right)=C_{facet}^{In}+\left(1-C_{facet}^{In}\right)\sqrt{z/H}$$

gives the indium distribution along the facets. The constants $C_{facet}^{In}=0.25\pm 0.06$ and $C_{base}^{In}=0.40\pm 0.06$ stand for the indium concentration in the facets and the base of the reference QD for z = 0.

Due to the stochastic nature of the self-organization of the InGaAs QDs, they slightly differ from each other in sizes and other parameters. The lateral and height size distributions of the whole array of QDs are typically close to normal [25]. These are, however, dependent values. The larger the QD, the higher it is. As a result, the aspect ratio of the referent QD is characteristic of the whole array of the QDs. Despite the wide size dispersion, the 3D distribution of indium within a QD is unlikely to differ from one QD to another. Indeed, it’s about QD overgrowth conditions, which have a similar effect on the entire range of pure InAs QDs that have been formed initially of different sizes but the same shape. The physical phenomena that occur when GaAs overgrows InAs QDs are indium segregation and the minimizing of the elastic energy of QDs. As a result of this consideration, the phenomenologically generated indium distribution can be viewed as a 3D extension of Muraki’s segregation model. In support of this argument, a similar description of 3D composition distribution has been found to accurately describe a variety of systems, including SiGe islands [26]. The range of standard error bars of the FEM model parameters fit well the standard deviation of the parameters of experimental QDs. Accordingly, it is acceptable to assume that data gathered from a few QDs that have been validated by the FEM model are still valid for the majority of QDs in the array.

In addition to the pyramidal QDs, we also take into consideration the InGaAs wetting layer, which is well visible in the TEM micrographs and the out-of-plane component of the strain maps (Figure 1b). Due to the indium segregation and intermixing, the indium distribution in the wetting layer is not trivial. It can be approximated by the following analytical expression [16]:

(3) $$f_{WL}^{In}\left(z\right)=\frac{A}{zB\sqrt{2\pi }}\exp\left[-\frac{{\left(\ln z-\mu \right)}^{2}}{2B^{2}}\right],$$

where the constants *A* = 0.69, *B* = 0.70, and *µ* = 1.25 were determined by the best fit of the experimental data [16].

With the calculated mechanical stress-strain field we have solved the problem of quantum mechanics for electrons and holes on the same mash in a single-band approximation. We solely focus on the ground state of electrons and holes localized in the QD. We do not study any phenomena related to the interband mixing, since it is not inherent to the single-band model. We take into account that the ground state of holes in a pyramidal InGaAs QD is predominately composed of the heavy-hole band. The model is validated by comparison with the results of the eight-band *k · p*-model calculations discussed in Ref. [18] for the case of InAs QDs with a certain pyramidal shape.

## 3. Results

**Figure 2:**
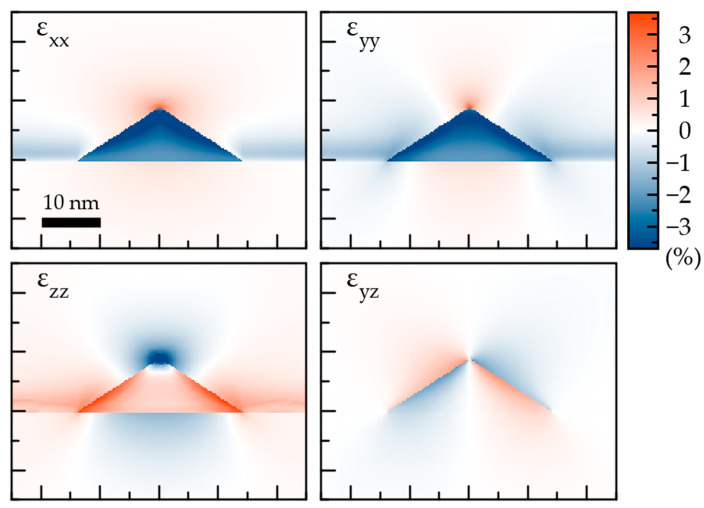
Elastic strain components calculated by FEM. Cross section of the reference InGaAs QDs is made by the ZY plane, passing through the symmetry axis of the pyramids. Central part of the cell is shown. Parameters of the QD: $L=28\mathrm{nm},H=9\mathrm{nm},C_{base}^{In}=0.40,C_{facet}^{In}=0.25$

Figure 2 shows the components of the elastic strain tensor calculated for the reference pyramidal QDs with the same aspect ratio and functional distribution of In as prescribed by Equations (1)–(3). The geometrical parameters and chemical composition of the QD exactly correspond to the reference values.

The $\varepsilon_{xx}$ and $\varepsilon_{yy}$ are negative over the whole cross-sections, which is a result of the InGaAs lattice distortion imposed by the GaAs substrate with a smaller lattice constant (see Table 1). Due to the positive Poisson’s ratio, the $\varepsilon_{zz}$ is positive. Near the top, it becomes more hydrostatic due to the geometrical reason. Higher indium concentration near the facets results in a larger elastic strain compared to the QD interior. The facets also produce a substantial shear component $\varepsilon_{yz}$. This component appears to be enhanced due to non-uniform indium distribution if we compare the results plotted in Figure 2 with similar calculations reported in Ref. [8]. The comparison of several QDs with different parameters reveals that the elastic strain map is very similar in both qualitative and quantitative sense.

The InGaAs wetting layer is included in the calculation as prescribed by Equation (3). Due to relatively low indium content, the wetting layer produces a relatively weak elastic tetragonal distortion. It does not produce any shear strain in the chosen geometry.

The non-uniform indium distribution and strain-stress field result in a specific profile of the localizing potential for electrons and holes. There are several different contributions to the potential. One of them directly comes from the composition dependence of the bandgap, which is displayed in Table 1. We assume the band offset to be 0.8 *E_g_* for electrons and 0.2 *E_g_* for holes. The compressive hydrostatic strain pushes up the electron potential in the QD.

(4) $$\delta E_{e}=a_{c}tr(\varepsilon ),$$

where the deformation potential *a_c_* is negative. The valence band is sensitive to all the components of the strain tensor. At the same time, the compressive hydrostatic component of the strain tensor reduces the localizing potential, the deviatoric and shear components of the tensor split the zones of heavy and light holes. This splitting in the center of the Brillouin zone can be described as [27]

(5) $$\delta E_{h}=a_{h}tr(\varepsilon )\pm \sqrt{b^{2}\left[{\left(\varepsilon_{xx}-\varepsilon_{yy}\right)}^{2}+{\left(\varepsilon_{xx}-\varepsilon_{zz}\right)}^{2}+{\left(\varepsilon_{zz}-\varepsilon_{yy}\right)}^{2}\right]/2+d^{2}\left(\varepsilon_{xy}^{2}+\varepsilon_{xz}^{2}+\varepsilon_{yz}^{2}\right)}$$

The parameters of the hole deformation potential are listed in Table 1.

**Figure 3:**
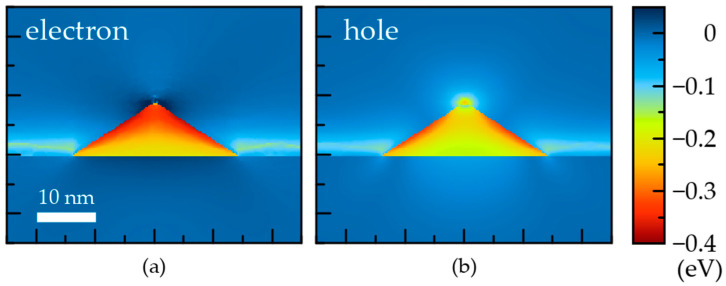
Localizing potentials for (**a**) electrons and (**b**) holes. A cross-section of the reference InGaAs QDs is made by the ZY plane, passing through the symmetry axis of the pyramids.

The maps of the localizing potentials are plotted in Figure 3 for electrons and holes in the reference QD. For electrons, the deepest potential is near the facets in the middle of them and in the upper part of the QD bulk. For holes, the localizing potential is even more concentrated near the QD facets. The piezoelectric field reduces the symmetry of the localizing potential from C_4v_ to C_2v_. Our calculations reveal that piezoelectricity’s contribution to the localizing potential is small compared to the chemical and strain contributions. In this paper, it was neglected, as in earlier works [8,18].

**Figure 4:**
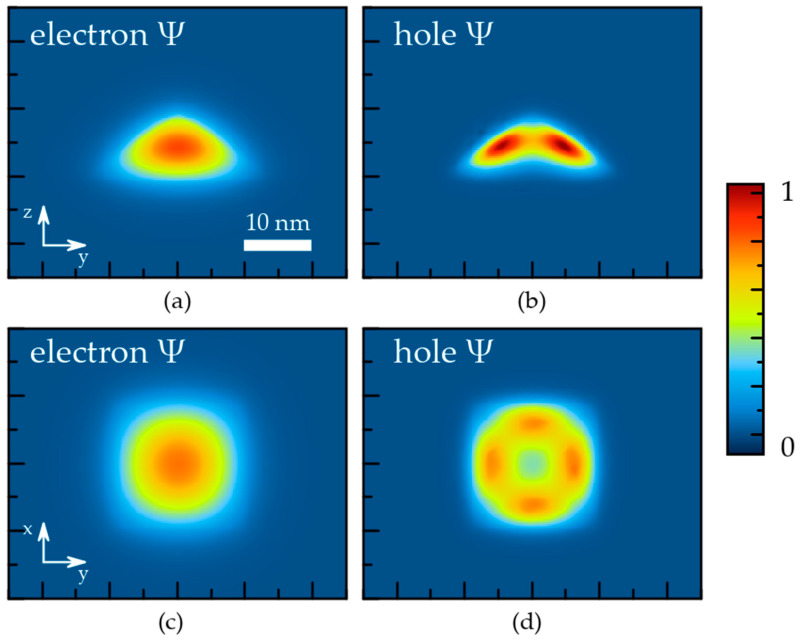
Wave functions of the ground states of (**a**,**c**) electrons and (**b**,**d**) holes. A cross-section of the reference InGaAs QDs is made by: (**a**,**b**) the ZY plane, passing through the symmetry axis of the pyramids. (**c**,**d**) the XY plane, passing 3 nm above the interface.

The determined localizing potentials are utilized in the quantum-mechanical problems, which are solved separately for the electrons and holes. The wave functions of the electron and hole ground states in the QD are plotted in Figure 4.

Though the localizing potential is deeper near the QD facets, the electron wave function is spotted in the QD bulk with the largest value almost in the middle of the symmetry axis. It is a result of the relatively small effective mass of the electrons. The effective mass of heavy holes is substantially larger (Table 1). Therefore, the hole wave function appears to be more sensitive to the potential profile. The hole wave function at the ground state does not have the largest value in the QD center, unlike the electron wave function. The probability is distributed rather as a bagel; however, it remains substantial near the QD center. The overlap integral for the electron and hole ground states appears to be 0.8, i.e., it is rather high.

## 4. Discussion

The modeling results described above are solely based on the structural data obtained experimentally for InGaAs QDs formed by a common technological procedure during the MBE process. Therefore, the model does not rely on any fitting parameters and utilizes a standard set of materials parameters listed in Table 1. It is worth comparing the calculated results with the experimental spectrum of the optical emission from the QDs. In the case of photoluminescence recorded at low temperature, the optical emission is mostly associated with radiative decay of the excitons formed by electrons and holes at the ground states in the QDs. The exciton binding energy depends on the QD parameters but can be roughly estimated as 10 meV.

**Figure 5:**
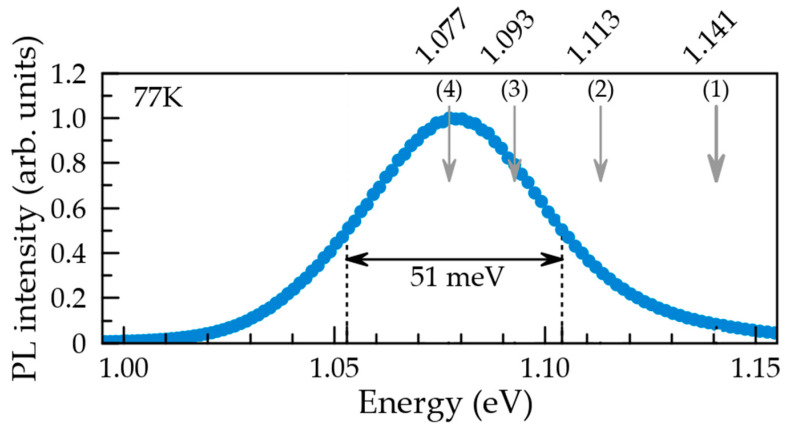
Experimental spectrum of the photoluminescence recorded at 77 K from the ensemble of the InGaAs QDs. Vertical arrows mark the emission of excitons from the QDs with the following parameters.

Figure 5 shows the experimental spectrum of photoluminescence (PL) of InGaAs QDs recorded at 77 K. It is important to emphasize that the structural and optical research was done on the same sample. The growth details for this sample can be found in Ref. [16].

The excitonic emission from a single QD provides a sharp line in the optical spectrum. Unfortunately, it is impossible to record the optical line and investigate the structural details for the same QD. Due to the spontaneous stochastic nature of the self-organization, different QDs in the ensemble have slightly different parameters. This includes the QD volume, shape, and total indium content. The dispersion of the QD parameters results in an inhomogeneous broadening of the PL band.

(1) $L=28\mathrm{nm},H=9\mathrm{nm},C_{base}^{In}=0.40,C_{facet}^{In}=0.25$;
(2) $L=30\mathrm{nm},H=10\mathrm{nm},C_{base}^{In}=0.45,C_{facet}^{In}=0.30$;
(3) $L=34\mathrm{nm},H=11\mathrm{nm},C_{base}^{In}=0.45,C_{facet}^{In}=0.30$;
(4) $L=34\mathrm{nm},H=11\mathrm{nm},C_{base}^{In}=0.50,C_{facet}^{In}=0.35$.

Vertical arrows in Figure 5 indicate the calculated energies of excitons at ground states for InGaAs QDs with various parameters. The reference QD (line 1) appears to be at the high-energy side of the band. If the indium distribution (Equations (1) and (2)) and the pyramid aspect ratio are preserved, the red shift of the emission can be provided by a slightly higher total indium content and by some increase in the QD volume. Lines 2, 3, and 4 show the impact of these parameters on the photon energy. The maximum of the experimental PL band corresponds to a pyramidal QD with 34 nm base, 11 nm height and indium concentration described by Equations (1) and (2) with the parameters $C_{base}^{In}=0.50,C_{facet}^{In}=0.35$. So, the variations of the parameters from those of the reference QD are quite reasonable. In fact, the dispersion of the InGaAs QD linear sizes is commonly from 10 to 20% [28]. Such dispersion scales over a wide range of the QD densities and other characteristics of the QD ensemble [25].

**Figure 6:**
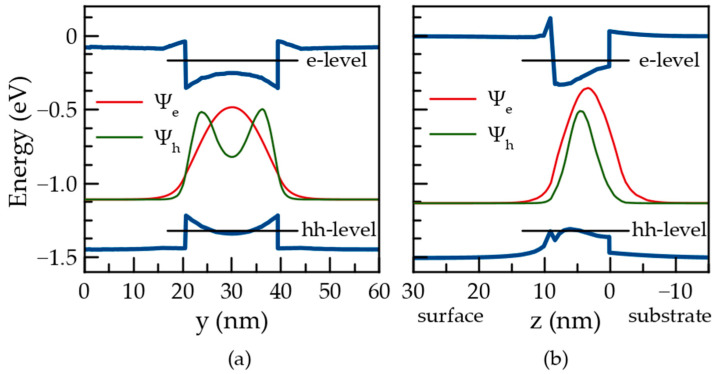
Localizing potentials for electrons and holes (blue solid lines), electron and hole wave functions (red and green lines, respectively), and ground levels of electrons and holes localized in the QD (black lines). The data are plotted along: (**a**) *y*-axis passing 3 nm above the interface; (**b**) *z*-axis passing the pyramid center.

The FEM calculations, which include the elasticity of the atomic system and quantum mechanics of the electron and hole systems, have previously been used for the description of the electron and hole eigenstates in self-organized epitaxial InGaAs QDs of variable shape and in different environments [7,8,18,29]. In such calculations, the indium concentration, $C_{QD}^{In}$, has commonly been assumed to be uniformly distributed, and its value is considered a fitting parameter. This assumption obviously contradicts the structural data (see Figure 1 and data in Refs. [6,16]). It also leads to inaccurate predictions of the ground state wave functions. In fact, the uniform in-plane indium distribution always results in the wave functions spotting near the QD center for both electron and hole ground states [7,18]. Figure 4 shows that such a shape of the wave function is justified for the electron ground state, but it is not for the hole ground state. Figure 6 shows in-plane and *z*-axis cuts of the electron and hole localizing potentials along with the corresponding wave function for the ground states. The black lines indicate the energy of the ground states of the electrons and holes. It is evident that the quantum confinement energy for an electron is substantially higher than any relief of the potential within the reference QD. However, in the case of holes the quantum confinement energy is smaller due to a larger effective mass (see Table 1). As a result, the hole at the ground state faces barriers being inside the QD. Consequently, the in-plane cut of the hole wave function in Figure 6 has a camel shape, which is a representation of the bagel shape in Figure 4.

In this paper, we solely focus on the ground states. It is clear, however, that the energy of the hole excited state is higher, and, therefore, less sensitive to the potential profile. In addition, for the symmetry reason, the wave function at the second state should have a node near the QD center. As a result, the shape of the hole wave function should be much less sensitive to the non-uniform indium distribution. So, it should approach the predictions presented in Ref. [18]. The quantum energy of an electron is above the potential profile even at the ground state.

The unusual bagel shape of the hole wave function at the ground state is important for many phenomena. For instance, the overlap integral of the electron and hole ground states is equal to 0.8 in the reference InGaAs QD and decreases with increasing QD size. It means that larger InGaAs QDs possess a substantially lower radiative decay and absorption rates of the excitons at the ground levels. This phenomenon should be taken into account for the development of QD lasers, light-emitting diodes, solar cells, and other optical and optoelectronic devices.

The bagel shape of the hole wave function should also impact the magnetic properties, such as the *g*-factor, and it should result in a specific anisotropy. In particular, the specific distribution of the electron and hole densities results in a built-in dipole moment. The calculations for a uniform indium distribution predict the hole wave function to be below that of the electron with respect to the growth direction [30]. A graded indium concentration decreasing from base to apex results in the opposite sign of the dipole moment [30]. Our experimentally-verified model with the indium concentration increasing from the base to apex and from the middle to facets of the pyramid results in almost zero built-in permanent dipole moment.

## 5. Conclusions

The results of modeling of the epitaxial self-assembled InGaAs QDs show that precise structural data are well consistent with the optical emission spectra recorded for the same sample. The utilized model, being quantitatively accurate in the calculations of the electron and hole ground state energy, predicts an unusual bagel-like shape of the hole wave function. It should cause a reduction in the electron-hole integral and changes in the optical and magnetic properties of such QDs.

## Figures and Tables

**Table 1:** Material parameters of the In_c_Ga_1−c_As solid solution for 77 K [23,24].

Quantity	Unit	Value
Lattice constant	nm	0.5653 (1 − *c*) + 0.6058 *c*
Band gap	eV	1.507 (1 − *c*) + 0.405 *c* − 0.475 *c*(1 − *c*)
CB effective mass	m_0_	0.067 − 0.044 *c*
VB effective mass	m_0_	0.51 − 0.1 *c*
CB hydrostatic def. pot. *a_c_*	eV	−8.013 + 2.933 *c*
VB hydrostatic def. pot. *a_v_*	eV	−0.220 − 0.780 *c*
VB deviatoric def. pot. *b_v_*	eV	−1.824 + 0.024 *c*
VB shear def. pot. *d_v_*	eV	−5.062 + 1.462 *c*
Elastic compliance *C*_11_	GPa	119 − 35.6 *c*
Elastic compliance *C*_12_	GPa	53.4 − 8 *c*
Elastic compliance *C*_14_	GPa	56.9 − 17.4 *c*
Static dielectric constant		12.89 + 1.35 *c* + 0.76 *c*^2^
Piezoelectric modulus *e*_14_	C m^−2^	0.16 − 0.11 *c*

## Data Availability

The data presented in this study are available on request from the corresponding author.
